# Census Parcels Cropping System Classification from Multitemporal Remote Imagery: A Proposed Universal Methodology

**DOI:** 10.1371/journal.pone.0117551

**Published:** 2015-02-17

**Authors:** Luis García-Torres, Juan J. Caballero-Novella, David Gómez-Candón, José Manuel Peña

**Affiliations:** Institute for Sustainable Agriculture, Spanish Council for Scientific Research (CSIC), Cordoba, Spain; University of Vigo, SPAIN

## Abstract

A procedure named CROPCLASS was developed to semi-automate census parcel crop assessment in any agricultural area using multitemporal remote images. For each area, CROPCLASS consists of a) a definition of census parcels through vector files in all of the images; b) the extraction of spectral bands (SB) and key vegetation index (VI) average values for each parcel and image; c) the conformation of a matrix data (MD) of the extracted information; d) the classification of MD decision trees (DT) and Structured Query Language (SQL) crop predictive model definition also based on preliminary land-use ground-truth work in a reduced number of parcels; and e) the implementation of predictive models to classify unidentified parcels land uses. The software named CROPCLASS-2.0 was developed to semi-automatically perform the described procedure in an economically feasible manner. The CROPCLASS methodology was validated using seven GeoEye-1 satellite images that were taken over the LaVentilla area (Southern Spain) from April to October 2010 at 3- to 4-week intervals. The studied region was visited every 3 weeks, identifying 12 crops and others land uses in 311 parcels. The DT training models for each cropping system were assessed at a 95% to 100% overall accuracy (OA) for each crop within its corresponding cropping systems. The DT training models that were used to directly identify the individual crops were assessed with 80.7% OA, with a user accuracy of approximately 80% or higher for most crops. Generally, the DT model accuracy was similar using the seven images that were taken at approximately one-month intervals or a set of three images that were taken during early spring, summer and autumn, or set of two images that were taken at about 2 to 3 months interval. The classification of the unidentified parcels for the individual crops was achieved with an OA of 79.5%.

## Introduction

### 1. Global importance of land-use classification

Policy makers are responsible for food security and land-use planning and require accurate and timely information on crop production at the regional level. Furthermore, crop identification on specific parcels and the assessment of soil management practices are also important when adhering to administration requirements. Traditionally, crop areas are reported based on census data, which cannot provide geographical distribution information. In addition, this process is tedious, time consuming and costly [1]. Agricultural land use information is, therefore, updated routinely in many cropland regions in the USA and Europe through farmer communication or ground visits by administrative inspectors to selected fields [2, 3]. The EU has developed the Land Parcel Identification System [4], which provides geo-referenced, on-line information that is supported by up-to-date nationwide image datasets. This system was designed as the main instrument for the implementation of the Common Agrarian Policy first pillar—direct payments to the farmer, i.e., to identify and quantify the land that is eligible for subsidy payment or for the implementation of agro-environmental measures payments.

### 2. Crop classification vs. remote sensing

Remote sensing, either alone or in combination with ground surveys, plays an increasingly important role in delivering accurate and timely information regarding the location and area of crop types, crop stress, productivity, and other relative variables, such as irrigation requirements [5, 6, 7]. Odenweller and Johnson (1984; [8]), using multitemporal Landsat images of the USA Corn Belt (USA), studied the temporal-spectral profile of a green vegetation indicator and noted the different profile through time of non-vegetation classes, of perennial vegetation and of specific annual crops, such as winter cereal and sunflower. Lobel and Asner (2004; [5]) estimated the surface of wheat and maize in a large area of Northwest Mexico and in the Southern Great Plains (USA) using Modis and Landsat and assessed the crop temporal evolution of Red, NIR (near-infrared) and PVI (= NIR-Red).

Very diverse methodological approaches have been used to achieve crop/cropping system classification using remotely sensed images. Usually, maps of cropland distribution are generated by analyzing remotely sensed data throughout the growing season and applying both soft and hard image classification methods [3, 6, 9, 10]. Unsupervised hard classification methods, such as K-mean, ISODATA and maximum likelihood classification (MLC), are commonly used [11] and can achieve good results in large homogenous areas where pure pixels are dominants, but they fail in fragmental areas where mixed pixels are dominant. Lobel and Asner (2004;[5]) demonstrated the importance of subpixel heterogeneity in cropland systems and the potential of temporal unmixing to provide an accurate and rapid assessment of the land cover distribution using low/coarse spatial resolution sensors. Generally, the use of satellite images of low/coarse resolution is limited by mixed pixels and can only provide an assessment of large parcels. For example, Modis imagery of 250 m of pixel can only be appropriated for parcels that are larger than 32 ha [12, 13]. Serra and Pons (2008; [14]) presented a methodology for mapping and monitoring the temporal signatures of six main Mediterranean crops (winter wheat, rice, corn, alfalfa, fruit trees and others) in central Spain using a hybrid classifier and Landsat images, reported that a multitemporal approach is essential and recommended the incorporation of phenology data in the classification methodology. Simoneaux et al. (2008) [15] used a time series of eight Landsat images over Morocco to identify four main classes (bare soil, annual vegetation, trees on bare soil and trees on annual understory), although these authors concluded that a precise typology of the crops could not be obtained based on the Normalized Difference Vegetation Index [NDVI = (NIR-R)/(NIR+R)] profiles. Zhong et al (2014) [16] developed a crop classification method that is based on spectral and phenological metric indices and that can be used in multiple years using training data of a single year.

The identification of improved classification methods, such as the Artificial Neural Network (ANN), Decision Tree (DT) and Support Vector Machine (SVM) [17, 18], has been a longstanding topic of research interest in cropping system classification using remote images. Some authors have recommended the use of non-parametric classifiers, such as DT, which can handle an information class with multiple subclasses to accommodate this intra-class variability [12, 13]. Peña-Barragán et al. (2011) [3], using object-based image analysis techniques and Aster satellite scenes, developed a methodology for the multi-seasonal assessment of 13 major crops that are cultivated in Yolo County, California, USA, concluding that NDVI was predominantly used to identify the main groups of crops based on the presence and vigor of green vegetation, contributing approximately 50% to the models.

### 3. Decision Trees as a statistical tool for land use/crop classification

A DT is defined as a connected, acyclic, tree-based classification model of an undirected graph, with a root node containing all of the data, zero or more internal nodes (splits), and one or more leaves or terminal nodes (leaves) [17, 19]. A DT is a non-parametric classifier that does not require any a priory statistical assumption regarding the distribution of the data. A DT is based in a multistage or hierarchical decision scheme of a tree-like structure. Each node of the decision tree structure makes a binary decision that separates either one class or some of the classes from the remaining classes. Further, the selection of the split variable value in each node is only dependent on the set of observations in that node, and not on all the observations of the dataset [16]. A DT classifies cases into groups or predicts the values of a dependent (target) variable based on the values of independent (predictor) variables. This procedure provides validation tools for exploratory and confirmatory classification analysis. The data are recursively divided down the decision tree according to the defined classification framework. At each node, a decision tree is required, and this can be implemented using univariate splitting if using a single attribute or multivariate splitting if using several attributes [19].

Classification using DT analysis is increasingly applied in remote-sensing data [18, 19, 20]. Investigations using DT have demonstrated successful performance in the use of remotely-sensed data for the analysis of general land cover [21], vegetation cover types [22], tropical forest types [23], and cropping systems [3]. A DT offers unique advantages compared to other classification approaches, such as SVM and MLC [24], including the interpretation of the modeled tree structure and of the achieved results. In addition, a DT is computationally fast and makes no assumptions regarding the distribution of the data.

### 4. General and specific objectives

The aim of this study was to develop a procedure named CROPCLASS to semi-automate census parcel crop/cropping assessment in any agricultural area using multitemporal remote sensing images. CROPCLASS aims to significantly reduce the ground-truth visits and the required monitoring to obtain and implement the predictive crop model based on the SB and VI. The specific objectives are 1) to semi-automate the census parcel definition; 2) to automatically extract the SB and VI for each parcel in each multitemporal image and conform the overall matrix data; 3) to perform a DT analysis of the matrix data to obtain crop/land use predictive models; 4) to use the predictive models to identify the crops/cropping systems in the unidentified/undetermined parcels; 5) to develop CROPCLASS-2.0 software to semi-automatically manage all the previous steps; and 6) to validate the CROPCLASS procedure and software using a series of multitemporal remote sensing images that were taken over an agricultural area.

## Materials and Methods

### 1. Study location and GeoEye-1 satellite image acquisition

Seven multi-spectral GeoEye-1 satellite images (GeoEye-1, 2012) were taken over LaVentilla area (province of Cordoba, Southern Spain) from April to October 2010; each scene was approximately 100 km^2^. The geographic coordinates in the upper-left corner of the images are X = 315206 and Y = 4186133 (Universe Transverse Mercator System, zone 30 North, datum WGS-84). This region has a Mediterranean climate and is characterized by hot, dry summers (daily maximum temperatures greater than 38°C) and cool winters (daily maximum temperature of 14 to 15°C). The rainfall ranges from 450 to 800 mm and occurs mainly from October to April, but herbaceous crops are irrigated during the summer season. The images were taken on April 9, May 1, May 23, June 20, July 9, August 22 and October 2 and were named T1 to T7, respectively. The image spatial resolution was 2.00 m pixel^-1^, providing information on the blue (B, 450–510 nm), green (G, 510–580 nm), red (R, 655–690 nm) and near-infrared (NIR, 780–920 nm) spectral bands (SB). The swath width was 15.2 km. The ground was predominantly flat, with average slopes of a 2.12% grade. The georeferencing accuracy of the GeoEye-1 images was improved using ground control points (GCPs) and image-to-image co-registration as previously described [25]. The series of images were radiometrically normalized through pseudo-invariant features using the ARIN procedure [26].

### 2. CROPCLASS procedure and CROPCLASS-2.0 software

**Fig 1:**
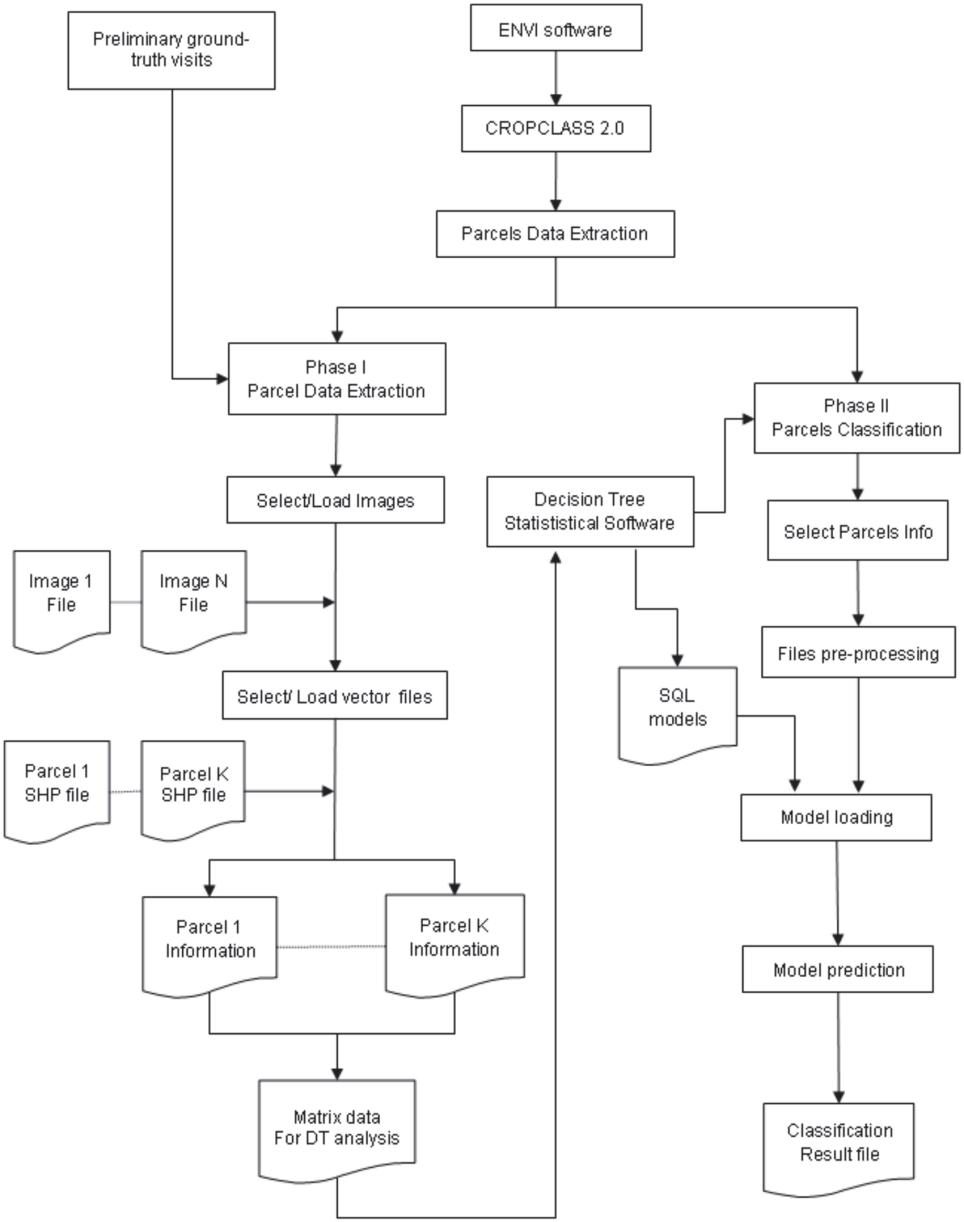
CROPCLASS procedure flowchart.

**Fig 2:**
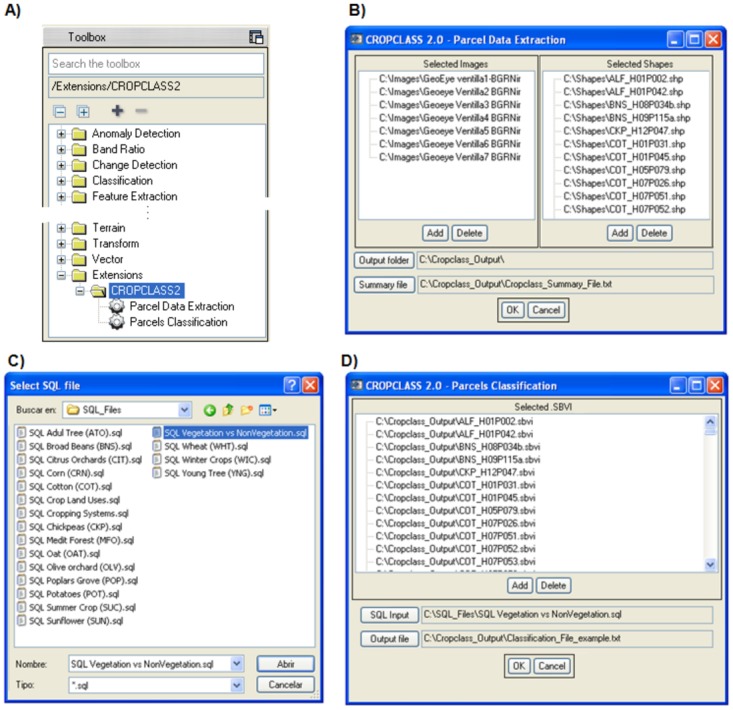
CROPCLASS-2.0 interfaces in ENVI-5.0. **A**: Toolbox→ Extensions → CROPCLASS 2.0. **B**: Selected images and vector parcels file loading; **C:** SQL predictive model files; **D:** Parcel classification process.

The CROPCLASS procedure requires a combination of image processing that is monitored through the CROPCLASS-2.0 software. This consist in using the spectral data extracted from each parcel of each image, the quality information from the identified parcels through ground-truth visits, and a Decision Trees (DT) analysis of the matrix data (Fig. 1). This analysis provides the land use/crop/cropping predictive models, which are required for the classification of the unidentified parcels. In our study, the Environment for Visualizing Images (ENVI-5.0, EXELIS- Visual Information Solutions, Boulder, CO, USA) software was used to visualize and process the images. The CROPCLASS-2.0 software [27] was developed as an add-on of ENVI-5.0 (Fig. 2A) to manage a multitemporal image series of the same geographical area.

**Fig 3:**
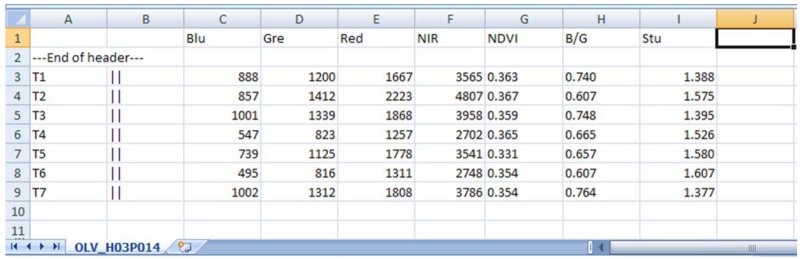
View of a spectral bands and vegetation index (SBVI) file. This was made by the CROPCLASS-2.0 software (phase I) for each parcel of each multitemporal image (T1 to T7).

**Fig 4:**
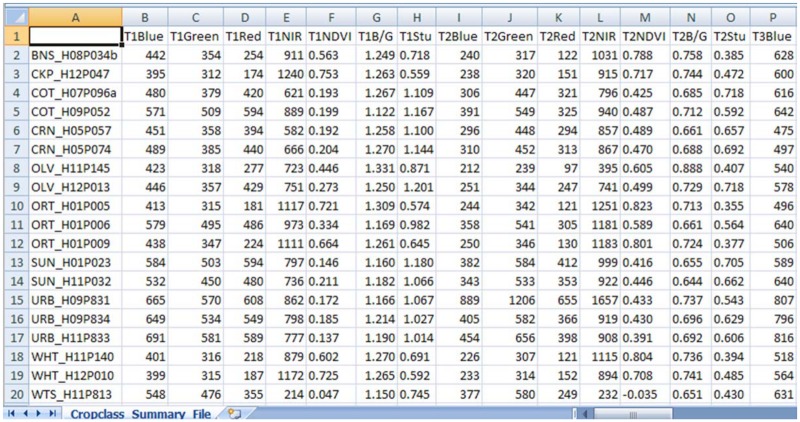
Partial view of a matrix data for the DT analysis.

CROPCLASS-2.0 acts in two phases, the first, named Parcel Data Extraction, semi-automatically achieves the following tasks: 1) loading multitemporal images and the vector files (.shp) into the corresponding parcels (Fig. 2B); 2) extracting the average spectral bands (SB) and estimating the selected vegetation index (VI) values for each parcel at each image (Fig. 3); and 3) conforming a SBVI file for each parcel, and a data matrix (DM) file for all parcels of a given geographical area scene. So that the DM includes the preliminary parcel ground-truth visit assessment on the land uses/ crops identified parcels (Fig. 4). The DM file is a text file that is ready to be loaded into the decision tree (DT) software, such as IBM SPSS, for analysis. Details of the DT analysis are explained later in this manuscript. The DT analysis of the DM provides, among other data, outcome the land use/ crop/ cropping system as a Structured Query Language (SQL, http://dev.mysql.com/downloads/mysql/) predictive models (PM) of the studied region (Figs. 1 and 2C; S1 Appendix). These models are used to classify the unidentified parcels through phase II of the CROPCLASS-2.0 software, which semi-automatically performs the following: 1) implementing the predictive models resulting from the DT analysis in each SBVI parcel files; 2) loading the SBVI files of the unidentified land-use parcels; and 3) yielding a classification output file with the predicted land uses/crops for each parcel (Fig. 2D; Table 1). All our data are original. All relevant data are within the paper. Data set and CROPCLASS-2.0 software for research purposes are available at the repository link http://dx.doi.org/10.5061/dryad.j958j.

**Table 1:** Partial view of the CROPCLASS-2.0-unidentified parcel classification output implementing diverse prediction model types.

	Model prediction types					
	NonVeg ^α^ -Veg		CropSys		Crop	
Parcel names						
	Land Use	Probability	Land Use	Probability	Land Use	Probability
CIV_H03P823.sbvi	NonVeg	1,00	WIC	0,95	BNS	0,25
CIV_H07P835.sbvi	NonVeg	1,00	YTO	1,00	BNS	0,25
CIV_H07_835Extra.sbvi	NonVeg	1,00	YTO	1,00	BNS	0,25
COT_H01P031.sbvi	Veg	1,00	SUC	0,90	COT	0,92
COT_H01P045.sbvi	Veg	1,00	SUC	0,90	COT	0,92
COT_H05P079.sbvi	Veg	1,00	SUC	0,90	COT	0,92
MFO_H04_842Extra.sbvi	Veg	1,00	ATO	0,93	MFO	0,89
MFO_H04_843Extra.sbv	Veg	1,00	ATO	0,93	MFO	0,89
MFO_H04_844Extra.sbvi	Veg	1,00	ATO	0,93	MFO	0,89
WHT_H01P004.sbvi	Veg	1,00	WIC	0,95	WHT	0,89
WHT_H01P008a.sbvi	Veg	1,00	WIC	0,95	WHT	0,89
WHT_H01P013.sbvi	Veg	1,00	WIC	0,95	WHT	0,89

^α^Abbreviations: NonVeg, non-vegetation; Veg: vegetation; CropSys, crop systems; BNS, broad beans; COT, cotton; MFO, Mediterranean forest; and WHT, winter wheat.

### 3. Field surveys and land-use identification

**Fig 5:**
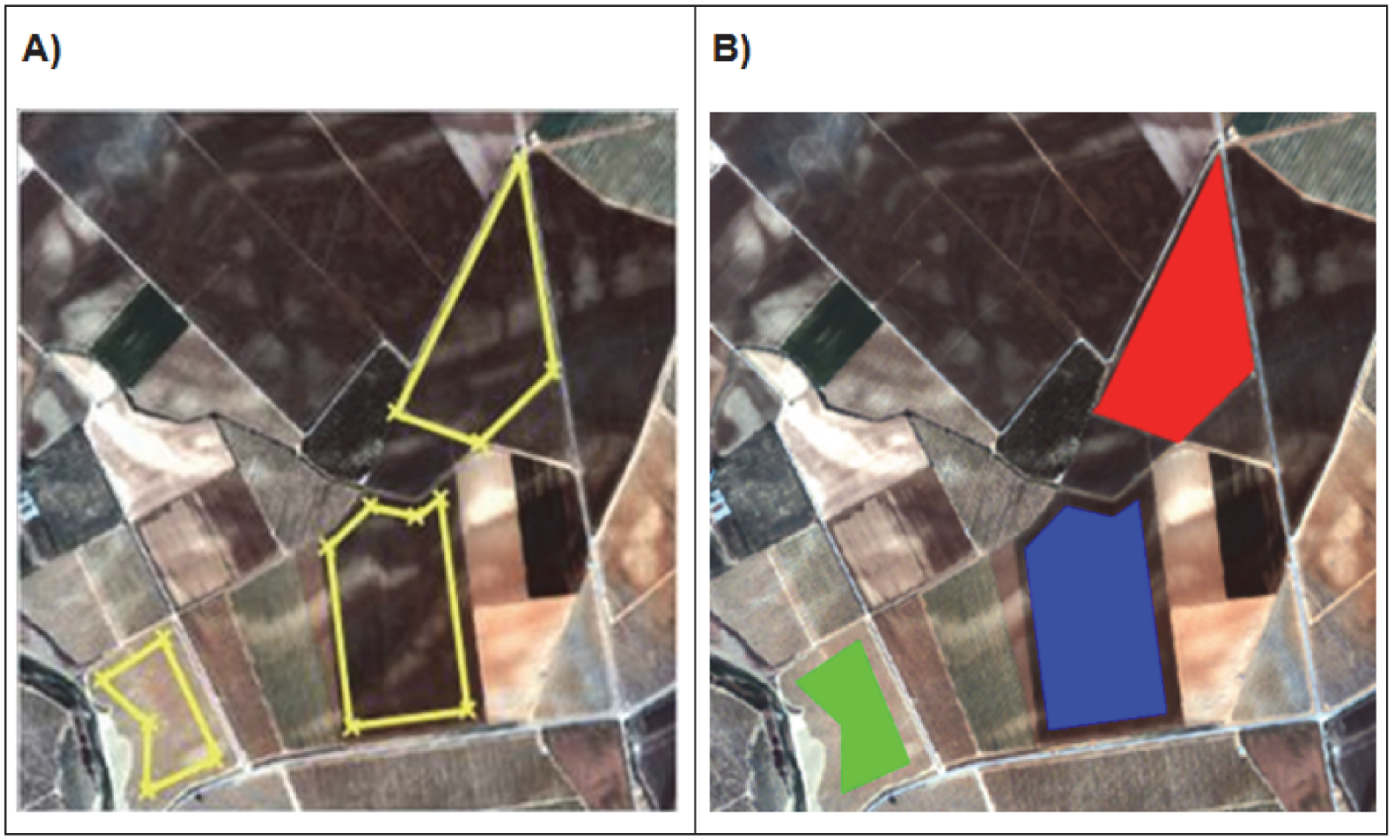
Partial view of a GeoEye-1 satellite image scene **A**) defining the parcel contour vertices; **B**) vector files completed.

The LaVentilla area was surveyed approximately every 3 weeks from April to October 2010 to identify the crop of each parcel, its stage of development, and any key agricultural feature, i.e., tree orchard spacing or cover crop (Fig. 5). A total of 311 parcels were recorded in the different visits, and twelve vegetative land uses were identified as follows: broad beans (BNS), chickpeas (CKP), cotton (COT), citrus orchards (CIT), corn (CRN), Mediterranean forest (MFO), oat (OAT), olive orchards (OLV), potatoes (POP), poplar grove (POP), sunflower (SUN), and winter wheat (WHT). Non-vegetative lands uses, such as bare soil/roads, civil buildings, flooded plots, paved roads, and water reservoirs were also found. No specific permission was required to survey our study area (LaVentilla, province of Cordoba, Spain). This is a free/ open access area by car, and the surveys were done “from the road”.

The main phenology features of each cropping system parcel throughout the growing season are shown in Table 2. Factors, such as the crop-planting pattern, growth stages, canopy structure and soil background critically influent the reflectance signal that is captured by sensor and the derived vegetation indices, thereby permitting crop identification in all types of parcels. The originally assessed crops and other land uses were merged into groups with seasonal and agronomic similarities during key growth periods to facilitate their discrimination (Tables 2 and 3). The herbaceous crops were grouped according to their main growing season: autumn-sown (winter crops, WIC) and spring-sown (summer crops, SUC). The adult tree orchards (ATO), specifically OLI, CIT and POP, which exhibited dense and permanent foliage throughout the study season, were differentiated from young tree orchards (YTO), where the vegetation cover was <30%. Similarly, roads, buildings, urban areas, rivers and water-flooded plots were considered for statistical analysis as a non-vegetative group.

**Table 2:** Phenology features in Southern Spain for the crops that were considered in this study.

	Jan.	Feb.	March	April	May	June	July	Aug.	Sept.	Oct.	Nov.	Dec.
BNS^α^	Veget.^β^	Veget.	Flower.	Fruit.	Senes.						Sowing	Emerg.
CKP	Veget.	Veget.	Flower.	Fruit.	Senes.						Sowing	Emerg.
CIT			Veget.	Veget.	Fruit.		Fruit.	Fruit.	Fruit.			
COT				Sowing.	Emerg	Veget.		Bloom.	Fruit.	Senes.		
CRN				Sowing.	Emerg	Veget.	FruitS.	Fruit.	Senes.			
MFO			Veget.	Veget.	Veget.	Fruit.	Fruit.	Fruit.	Fruit.	Fruit.		
OAT	Veget.	Veget.	Flower.	Fruit.	Fruit.	Senes.	Senes.				Sowing	Emerg.
OLV			LeafG.	LeafG.	LeafG.	Fruit.	Fruit.	Fruit.	Fruit	Fruit.	Fruit.	
POT					Sowing	Emerg.	Bloom.	Senes.	Senes.			
POP			LeafG.	LeafG.	LeafG.		Fruit.	Fruit.	Fruit.	Senes.	Senes.	Senes.
SUN			Sowing	Emerg.	Veget.	Flower.	Senes.	Senes.				
WHT	Veget.	Veget.	Flower.	Fruit.	Senes.						Sowing	Emerg.

^α^ Crop abbreviations: BNS, broad beans; CKP, chickpeas; CIT, citrus orchards; COT, cotton; CRN, corn; MFO, Mediterranean forest; OAT, oat; OLV, olive orchards; POP, poplars grove; POT, potatoes; SUN, sunflower; WHT, winter wheat.

^β^ Growth stages abbreviation: Veget., vegetation; Flower., flowering; Bloom., blooming; Senes., senescence; Fruit., fruiting; Emerg., emergence; LeafG., leaf growing

**Table 3:** Crops and other land uses that were assessed in the field survey and their groupings as dependent variables for the decision tree analysis.

All land uses	Cropping systems & others	Crops & others
Vegetation (Veg)		CIT ^**α**^
	Adult trees orchards(ATO)	MFO
		OLV
		POP
		COT
	Summer crops (SUC)	CRN
		POT
		SUN
		BNS
	Winter crops (WIC)	CKP
		OAT
		WHT
	Young orchards (YTO)	YTO
Non-Vegetation (Non-Veg)	Civil work (CiWo)	Buildings/ urban areas, pavement roads
	Water surface (WTS)	Flooded plots, water, reservoir, river

^α^ Abbreviations: BNS, broad beans; CKP, chickpeas; CIT, citrus orchards; COT, cotton; CRN, corn; MFO, Mediterranean forest; OAT, oat; OLV, olive orchards; POP, poplars grove; POT, potatoes; SUN, sunflower; WHT, winter wheat.

### 4. Spectral bands (SB) and vegetation index (VI) evolution

**Fig 6:**
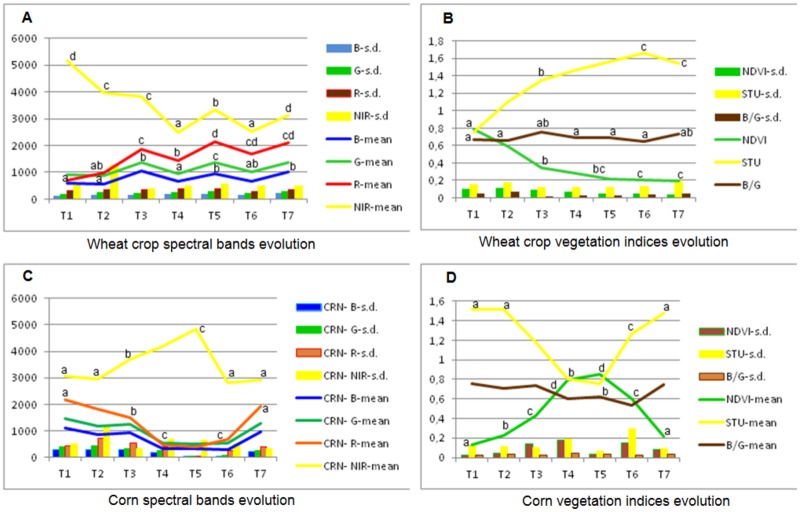
Spectral bands’ digital values and vegetation indices’ average evolution in the wheat (A & B) and corn (C & D) parcels. The vertical bars are standard deviations (s. d.). In the abscissa is the remote image timing (T1- early April to T7- early October, approximately one-month intervals); the data are presented as the means, and the vertical bars represent the standard deviation (s.d.) of six parcels of approximately 3 ha each. For each land use, the average data of the images followed by a different letter are significantly different at P≥0.05.

**Fig 7:**
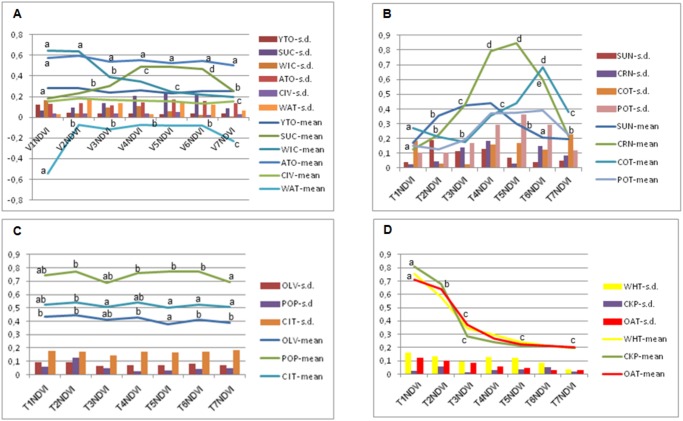
NDVI average evolution of: A) main cropping systems and land uses; B) summer crop; C) trees orchards; and D) winter crops. Vertical bars are standard deviations (s. d.). In abscissa is the remote image timing (V1- early April to V7-early October, about one month interval). Abbreviations as in Tables 2 and 3. Data are presented as the means, and vertical bars represent the standard deviation (s. d.) of six parcels of approximately 3 ha each). For each land use, the average data of the images followed by a different letter are significantly different at P≥0.05.

In our validation study, the four SB and three VI indices were used: the normalized difference vegetation index NDVI, Stubble Index (Stu = R/G), and B/G, which are each widely used to identify agrarian land uses, such as vegetation, stubble/crop yellowing, and bare soil, respectively [3,28,29]. To illustrate the evolution of the SB and VI of the vegetative/non-vegetative land uses throughout the growing season, the average evolution of five wheat and six corn parcels, representing the winter and summer crops, respectively, was used to establish a data analysis strategy and DT results interpretation (Fig. 6). Similarly, the NDVI evolution of the main cropping systems and of the summer, winter and adult trees plantation crops throughout the growing season is key information for crop grouping decisions and result interpretation (Fig. 7).

### 5. Decision tree modeling and model evaluation

The model was designed using a training/validation/testing dataset procedure. First, a set of training/validation parcels were used to create and evaluate the decision trees and the land use prediction models. Then, a set of testing parcels were classified by implementing the prediction models that were previously determined, and the accuracy of this classification was measured using the confusion matrix method [30]. In our study, fifty percent of the parcels (156 out of 311) were used for the training and validation of the predictive models (10%-fold cross validation), and the other half (155 parcels) were used to validate the parcel classification resulting from implementing the predictive models.

The IBM SPSS Statistics-21 software was used for the classification and regression decision tree (DT) analysis (IBM North America, New York, NY, United States). The CRT DT split the data into segments that were as homogeneous as possible with respect to the dependent/ response variable. The categorical dependent variables were the land use and crop groups as shown in Table 3. The DT node-splitting rule was the Gini index, which is a measure of impurity for a given node, and its application attempts to maximize the homogeneity of the child nodes with respect to the values of the dependent variable [19, 20]. The Gini index reaches its minimum (zero) when all of the cases in a node fall into a single category, which is the default measure. The difference between two consecutive nodes’ impurity is the splitting improvement. The Gini index is described by the equation

$$Gini\;(t)\;=\;\sum_{i}^{}pi\;(1-pi)$$

where pi is the relative frequency (determined by dividing the total number of observations of the class by the total number of observations) of class *i* at node *t*, and node *t* represents any node (parent or child) at which the given data are split. The CRT DT analyses were performed using a tree growth limit of 5 and a minimum number of cases for each of the parent nodes of 8 and for the child nodes of 4. The DT model was evaluated using a cross validation (10%-fold) procedure. The classification accuracy was determined by applying the confusion matrix [30]. We used UA (user’s accuracy, % correctly classified parcels) and PA (producer accuracy) as complementary statistical (Tables 4 and 5). User’s accuracy (% of success) is opposite to commission error (% error) and producer’s accuracy is opposite to omission error. The risk estimate and its standard error is a measure of the tree’s predictive accuracy. For categorically dependent variables, the risk estimate is the proportion of cases that are incorrectly classified after adjustment for the prior probabilities. The total number of parcels that were assessed in each DT analysis is indicated in the results section.

**Table 4:** Evaluation of the DT training models for the discrimination of adult tree orchards (ATO), summer crops (SUC) and winter crops (WIC).

ATO							SUC							WIC						
Observed/ predict.	MFO ^**α**^	OLV	ORT	POP	Predic	UA ^**β**^ (%)	Observed/ Predict	COT	CRN	POT	SUN	Total predict.	UA (%)	Observed/ predict	BNS	CKP	OAT	WHT	Total predict	UA (%)
MFO	8	0	0	0	8	100	COT	11	0	0	0	11	100	BNS	8	0	0	0	8	100
OLV	0	18	0	0	18	100	CRN	0	9	0	0	9	100	CKP	0	8	0	0	8	100
ORT	0	0	13	0	13	100	POT	0	0	5	0	5	100	OAT	0	0	5	0	5	100
POP	0	0	0	8	8	100	SUN	0	0	0	10	10	100	WHT	0	1	0	26	27	96
Total GTP	8	18	13	8	47			11	9	5	10	35			8	9	5	26	48	
PA (%)	1.0	1.0	1.0	1.0				1.0	1.0	1.0	1.0				100	88	100	100		
OA (%)						1.0						1.0								97
Tree Risk^**$**^	0.06	±0.03						0.07	±0.02						0.06	±0.03				

^**α**^Abbreviations: BNS, broad beans; CKP, chickpeas; CIT, citrus orchards; COT, cotton; CRN, corn; MFO, Mediterranean forest; OAT, oat; OLV, olive orchards; POP, poplars grove; POT, potatoes; SUN, sunflower; WHT, winter wheat.

^**β**^Statistical: UA, user accuracy (% correctly classified parcels); PA, producer accuracy; OA: overall accuracy;

^**$**^Tree risk: Estimated ±standard error.

For each DT analysis, the percentage of correctly classified land uses/parcels and the tree development risk were recorded. In addition, the number and the SQL (Structure Queries Language) models of each terminal node were also recorded. The standard SQL rules were generated to select/extract records from the database. The generated SQL rules save the case selection or scoring rules in an external file and then apply these rules to a different data source. The rules are based on the selected nodes in Tree Editor (IBM SPSS Statistics-21). The generated case-selection rules or scoring rules are in command syntax or SQL format. The tree risk estimate is a measure of its predictive accuracy, which includes the parcels that were incorrectly classified after adjustment for the prior probabilities. The SQL models of each terminal node were determined and stored in the CROPCLASS-2.0 software and used as predictive tools for the testing set of parcels.

### 6. Required image number and image-taking timing

To select the required number of images and the most adequate image-taking time to build DT models for crop/cropping system classification, several scenarios were studied as follows: seven images that were taken at approximately 3- to 4-week interval throughout the growing season from early spring (T1) to early autumn (T7), three images that were taken at T1, T4-early summer and T7, two sets of two images (T1–T4-early summer, T3-late spring and T6-late summer), and only one image at specific timings (T1 to T7). The CRT DT analysis was conducted as previously described.

## Results and Comments

### 1. Evolution of crop spectral bands and vegetation indices

The SB and VI indices of any herbaceous cropping system considerably varied throughout the growing season (Figs. 6 and 7). For example, the wheat NIR values decreased, and the R increased drastically from early spring (T1) to early summer (T4) because this is approximately the time winter crops are normally harvested, and the straw remained on the soil (Fig. 6). Accordingly, the wheat NDVI drastically decreased from early spring to summer, while the STU, expressive of crop senescence, increased during the same period; the same pattern was observed for the other winter crops (Fig. 6). Moreover, the wheat B/G index, indicative of the presence of bare soil remain, did not change much, expressing a high soil cover throughout the growing season. The opposite pattern occurred for herbaceous summer crops, such as corn: the R values decreased, and the NIR increased from early April (T1) to mid-August (T4). From T1 to T4 in the summer crops, the NDVI increased, after which NDVI drastically decreased, coinciding with the crop growing and senescence, respectively (Fig. 7).

**Fig 8:**
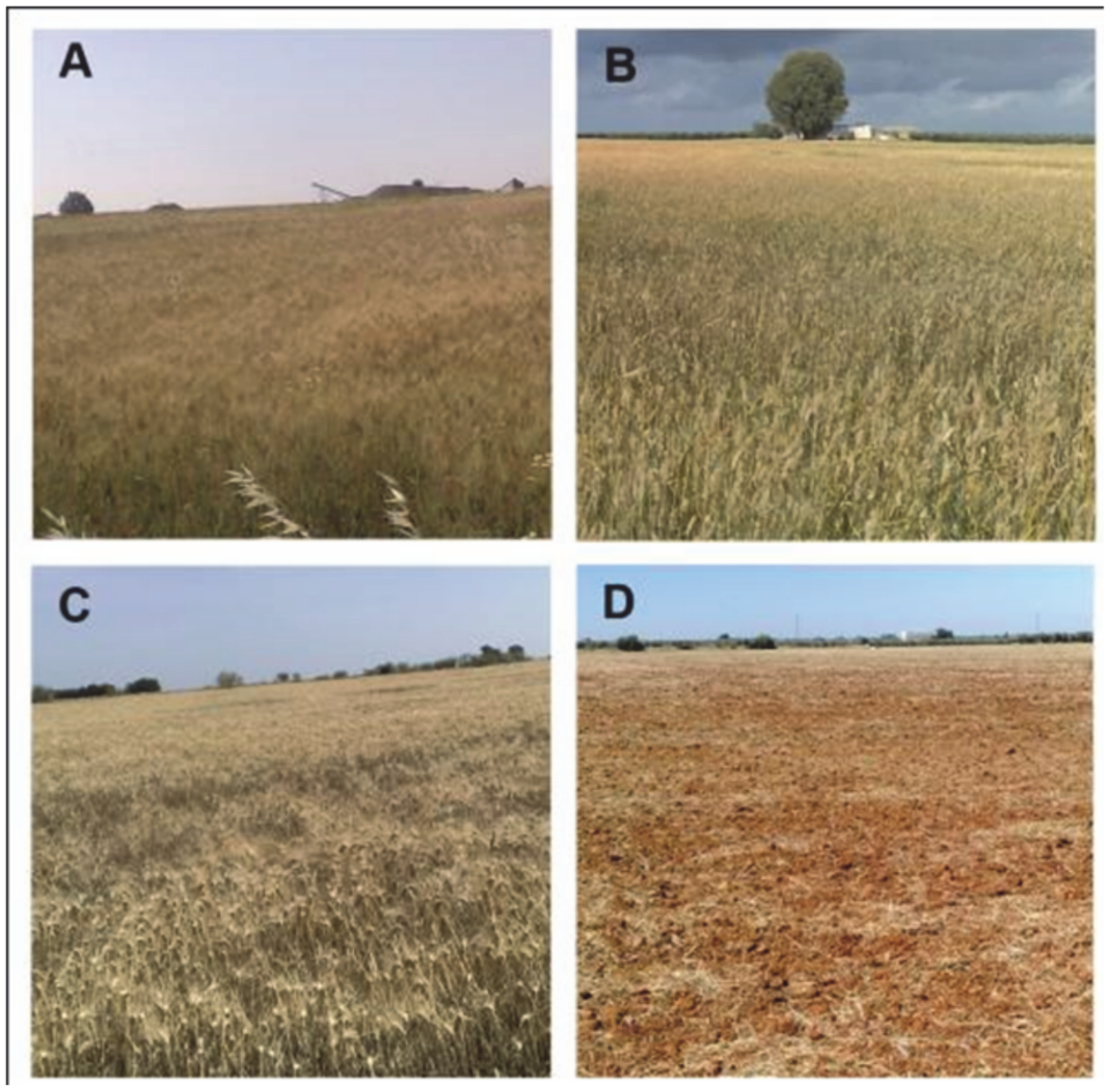
Wheat crop evolution. A: early April, grain-filling growth stage showing intense green color and therefore high NDVI values; B: early May, mid-senescence, green-yellowish color, and mid-NDVI values; C: late May/ early June, late senescence, predominant yellow color, low NDVI values; D: stubble, typical of mid-June throughout the summer. These growth stages roughly coincide with the T1, T2, T3 and T5 satellite images that were taken, and with the wheat NDVI data evolution as shown in Fig. 6d. The data are presented as the means, and the vertical bars represent the standard deviation (s. d.) of six parcels of approximately 3 ha each.

**Fig 9:**
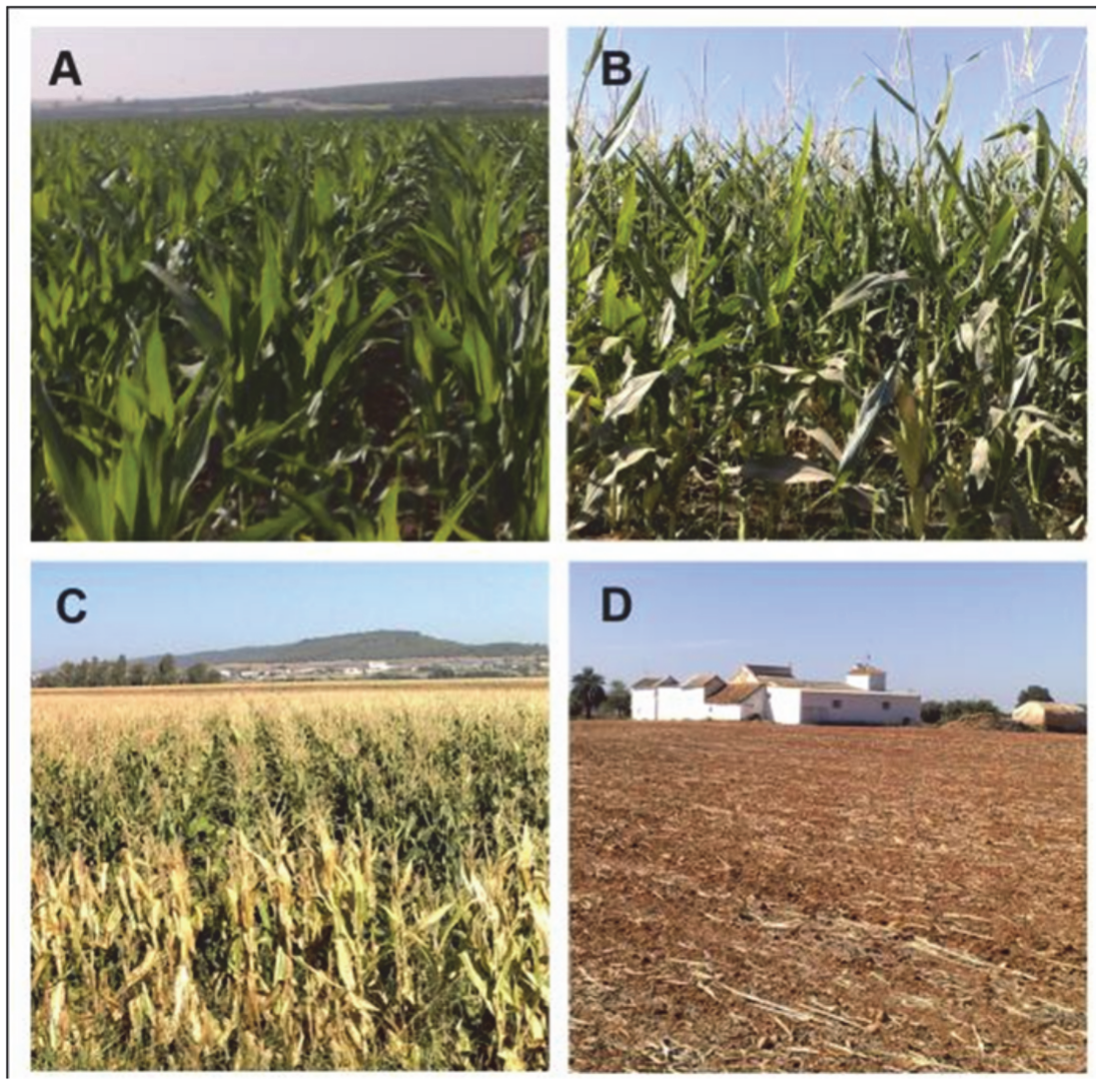
Corn crop evolution. A: late May and early June, corn during the vegetative growing phase, characterized by increasing NDVI values and coinciding with the T3–T4 satellite images of this stage; B: flowering stage, July, T5 image; C: senescence period, which take place in the second part of August (NDVI values decrease; satellite image T6); and D: corn stubble, beyond mid-September (satellite image T7). The corn NDVI data evolution is shown in Fig. 7B.

**Fig 10:**
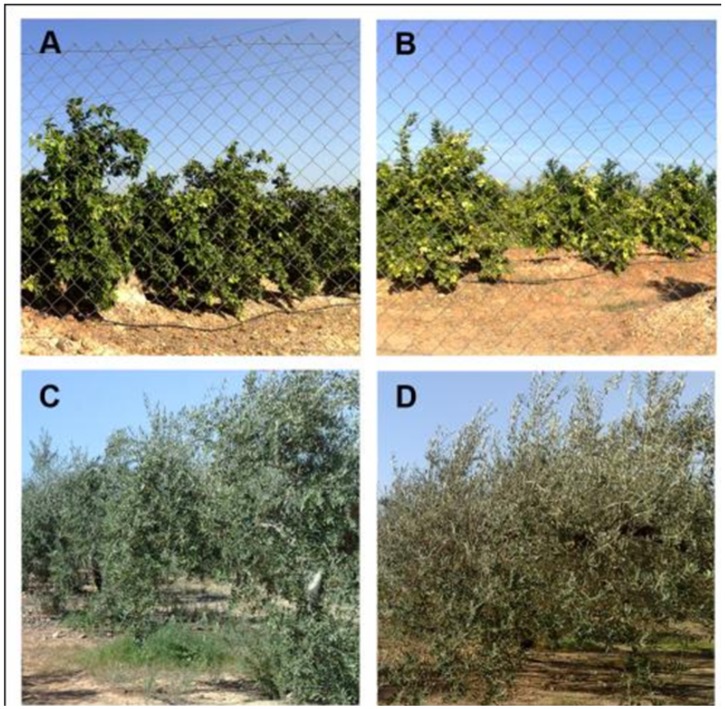
Canopy structure of citrus (A and B) and olive (C and D) orchards vary very little throughout the year, particularly during the main growing season (April to October). Photographs A and C were taken in early May, and B and D were taken in late September, roughly coinciding with the T2 and T7 satellite images of this study. In photograph D are the orange fruits but not the olives in photograph D at the distance where the photograph was taken. Due to the few changes in the tree canopy and covered soil surface, the NDVI values of citrus and olive are very similar throughout the growing season (Fig. 7C) and can be used as pseudo-invariant features for radiometric normalization [26].

The vegetative cropping systems, such as herbaceous winter and summer crops, adult tree plantations and young orchard plantations showed considerable differences in the NDVI evolution throughout the growing season between each other and when compared to that of the non-vegetative land uses (Fig. 7). For example, the NDVI for winter crops drastically decreased in spring from early May to June, and the opposite trend occurred for the summer crops. Consistent differences in the NDVI evolution were found in some of the summer crops, including sunflower, corn and cotton (Fig. 7), which can be partly attributed to the sowing dates, generally in March for sunflower and in mid-late April for corn and cotton. Adult tree orchards, such as those of citrus and olives, and poplar and Mediterranean forest plantations exhibited uniform NDVI values throughout the study season. The higher values of poplars trees and citrus in comparison to those of olives and Mediterranean forest are explained by the higher vegetation density and lower bare soil coverage. The winter crops’ NDVI pattern evolutions are somewhat similar to each other (Fig. 7), making discrimination more difficult within this vegetation index. The evolutions of the phenology pattern of wheat, corn and trees orchards are also illustrated in Figs. 8, 9 and 10, respectively.

### 2. DT training model evaluation

**Fig 11:**
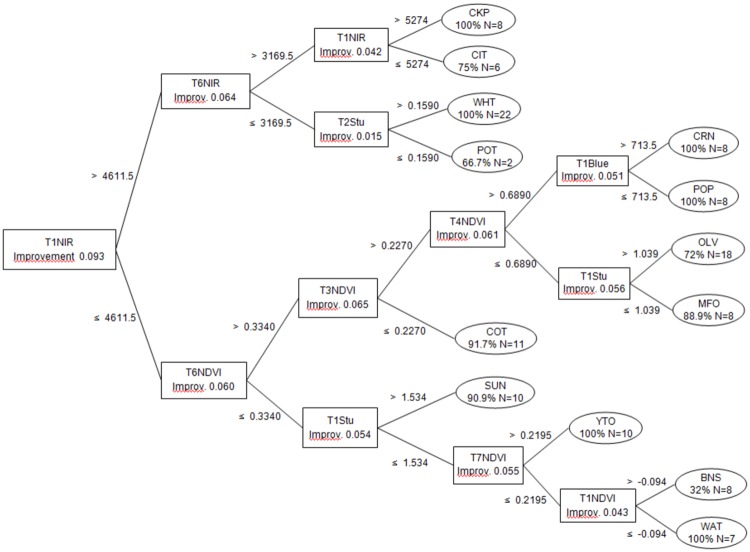
Decision tree training model. For each splitting decision, the key spectral bands and/or vegetation indexes, corresponding values and statistical improvement are indicated. The terminal nodes show the identified crop, number of parcels and user accuracy.

The CRT DT classification at the parcel level as affected by the set of used independent predictors, including the spectral bands and selected vegetation indexes, is shown in Tables 4 and 5 and Fig. 11. The vegetation parcels can be distinguished from the non-vegetation parcels with a 100% overall accuracy OA). Cropping systems such as SUC summer crops, winter crops and adult tree plantations can be distinguished at an approximately 97% or higher OA and a tree risk around 0.2±0.06 (Table 4). For the individual crop training model evaluations OA was 80.7% and tree risk 0.4±0.04 (Table 5). The individual crop user accuracy (UA) was approximately 80% higher for most crops, except for those of OAT, CIT, and POT, which were 50%–60%, likely due to the morphological pattern similarities of these crops with others, including OAT and WHT.

**Table 5:** Evaluation of the DT training models for the discrimination of individual crops and others land uses.

Observed	BNS^**α**^	CIT	CIV	CKP	COT	CRN	MFO	OAT	OLV	POP	POT	SUN	WAT	WHT	YTO	Obs. Parcels	UA ^**β**^ (%)
BNS	8	0	0	0	0	0	0	0	0	0	0	0	0	0	0	8	100
CIT	0	6	0	0	0	0	1	0	6	0	0	0	0	0	0	13	46
CIV	6	0	0	0	0	0	0	0	0	0	0	0	0	0	0	6	0
CKP	0	0	0	8	0	0	0	0	0	0	0	0	0	0	0	8	100
COT	0	0	0	0	11	0	0	0	0	0	0	0	0	0	0	11	100
RN	0	0	0	0	1	8	0	0	0	0	0	0	0	0	0	9	89
MFO	0	0	0	0	0	0	8	0	0	0	0	0	0	0	0	8	100
OAT	5	0	0	0	0	0	0	0	0	0	0	0	0	0	0	5	0
OLV	0	0	0	0	0	0	0	0	18	0	0	0	0	0	0	18	100
POP	0	0	0	0	0	0	0	0	0	8	0	0	0	0	0	8	100
POT	3	0	0	0	0	0	0	0	0	0	2	0	0	0	0	5	40
SUN	0	0	0	0	0	0	0	0	0	0	0	10	0	0	0	10	100
WAT	0	0	0	0	0	0	0	0	0	0	0	0	7	0	0	7	100
WHT	2	2	0	0	0	0	0	0	0	0	1	0	0	22	0	27	81
YTO	1	0	0	0	0	0	0	0	1	0	0	1	0	0	10	13	77
Predicted parcels	25	8	0	8	12	8	9	0	25	8	3	11	7	22	10		
PA (%)^**β**^	32	75	0	100	92	100	89	0	72	100	67	91	100	100	100		
OA (%)^**β**^																80.7	
Tree Risk^**$**^																0.4±.0.04	

^**α**^Abbreviations: BNS, broad beans; CKP, chickpeas; CIT, citrus orchards; COT, cotton; CRN, corn; MFO, Mediterranean forest; OAT, oat; OLV, olive orchards; POP, poplars grove; POT, potatoes; SUC, summer crops; SUN, sunflower; WHT, winter wheat; WIC, winter crops; YTO, Young Trees Orchards; GTP: ground truth parcel;

^**β**^Statistical: UA, user accuracy (% correctly classified parcels); PA, producer accuracy; OA, overall accuracy;

^**$**^Tree risk: estimated ±standard error.

### 3. Effect of image number and time in the CRT DT training models

Generally, the percentage of correctly classified parcels was similar using seven images that were taken at approximately one-month intervals (T1 to T7), three images that were taken at T1, T3 and T5 or at T2, T4, and T6, or two images that were taken at T2 and T5, or at T3 & T6 (Table 6). These results were 81%, 80.5% and 80.5%, respectively. Considering just one image, the average percentage of correctly classified parcels varied according to the image timing, ranging from 53% at T3 to 82% at T5, on average 69%. The CRT DT risk increased as the number of images decreased. For example, CRT DT risk was 0.23, 0.38 (average of two 3× images), 0.42 (average of two 2× images) and 0.50 (average of seven 1× images) (Table 6). Therefore, the DT tree and DT training models are less accurate, as the number of images that were taken decreases throughout development.

**Table 6:** Percentage of correctly classified parcels for all of the crops/land uses as affected by the number and timing of the image.

	No. parcels	T1 to T7 ^**α**^	T1, T3, T5	T2, T4, T6	T2, T5	T3, T6	T1	T2	T3	T4	T5	T6	T7	Mean T1–T7	Mean Overall
BNS ^**β**^	8	100	0	100	0	75	0	100	0	63	75	0	75	45	49
CIT	13	46	46	100	100	100	31	62	77	85	100	85	100	77	78
CIV	6	0	67	100	100	83	100	100	83	100	100	100	67	93	83
CKP	8	100	100	100	75	100	100	100	0	100	75	100	50	75	83
COT	11	100	91	100	73	100	0	64	100	27	64	64	0	45	65
CRN	9	89	100	67	100	33	89	67	0	89	89	44	0	54	64
MFO	8	100	0	100	100	100	100	100	100	100	100	100	100	100	92
OAT	5	0	0	60	100	60	0	60	0	40	40	0	0	20	30
OLV	18	100	100	94	67	100	100	83	0	83	61	94	83	72	81
POP	8	100	100	100	0	75	0	75	100	50	75	75	100	68	71
POT	5	40	0	60	0	0	0	0	0	60	0	0	0	9	13
SUN	10	100	100	100	100	100	80	80	80	80	100	100	80	86	92
WAT	7	100	0	100	100	100	100	43	100	100	100	100	100	92	87
WHT	27	82	93	78	96	82	78	59	96	67	96	82	78	79	82
YTO	13	77	100	85	92	69	69	69	0	92	92	69	54	64	72
OA ^**$**^		81	71	90	78	83	62	71	53	76	82	74	65	69	74
Mean		76	60	90	74	78	56	71	49	76	78	68	59	65	69
Tree risk^**٭**^	Estimat.	0.23±	0.40±	0.36±	0.34±	0.41±	0.43±	0.60±	.64±	0.52±	0.37±	0.52±	0.45±		
	s.e.	0.041	0.039	0.038	0.038	0.04	0.04	0.04	0.04	0.04	0.04	0.04	0.04		

^**α**^Image taking time from early April (T1) to October (T7) at about one month interval

^**β**^Abbreviations: BNS, broad beans; CKP, chickpeas; CIT, citrus orchards; COT, cotton; CRN, corn; MFO, Mediterranean forest; OAT, oat; OLV, olive orchards; POP,

Poplars grove; POT, potatoes; SUC, summer crops; SUN, sunflower; WHT, winter wheat; WIC, winter crops;

^**$**^OA overall accuracy;

^**٭**^Tree risk, estimated±standard error;

### 4. Selected independent/ predictor variables

For the decision trees analysis, four spectral bands (SB) and three vegetation indexes (VI) per each of the seven multispectral images were used, totaling forty nine independent variables. The most important independent variables varied widely with the considered cropping systems and with the image number and timing (Table 7). Indistinctly, the indexes or the spectral were more relevant than the other as independent variable. For example, to distinguish between the considered cropping systems, T6Blue, T6Red, T6NDVI and T3Gree exhibited a normalized importance that was greater than 90%, and for the summer crops were T3NDVI, T4Gree, T4Blue and T4Red. STU and NDVI, NDVI and Stu, and NIR and NDVI, were the two most influent independent predictors for T1, T4 and T7, respectively.

**Table 7:** Six selected independent variables for the diverse DT training analysis studies (in parentheses % of normalized importance).

Land uses/ Cropping Systems					Number and timing of image (T1 to T7) ^**α**^												All land uses
Veg ^**β**^–Non-Veg.	CropSys	WIC	ATO	SUC	T1 to T7	T1, T3, T5	T2, T4, T6	T2, T5	T3, T6	T1	T2	T3	T4	T5	T6	T7	
T4NDVI (100)	T6Blue (100)	T6Red (100)	T5NIR (100)	T3NDVI (100)	T5Gree (100)	T5NDVI (100)	T4NDVI (100)	T5Red (100)	T3NDVI (100)	Stu (100)	Gree (100)	TStu (100)	NDVI (100)	NIR (100)	BG (100)	NIR (100)	T3Stu (100)
T4B/G (100)	T6Red (97)	T5Blue (94)	T6NIR (88)	T4Gree (97)	T5Red (100)	T3Red (94)	T2NDVI (92)	T5NIR (99)	T3Red (90)	NDVI (88)	BG (100)	NDVI (95)	Stu (97)	Red (91)	Stu (97)	NDVI (99)	T1NDVI (98)
T3NDVI (72)	T6NDVI (94)	T6Blue (89)	T3Stu (79)	T4Blue (94)	T6Gree (97)	T1NDVI (92)	T4Blu (90)	T2NIR (91)	T6Stu (87)	Red (83)	Blue (89)	NIR (65	BG (94)	Gree (88)	NIR (90)	Stu (98)	T7Stu (97)
T7NDVI (63)	T6Gree (90)	T6Gree (89)	T7NIR (78)	T4Red (90)	T6Red (97)	T1Blue (91)	T4BG (87)	T5Stu (89)	T3Stu (85)	Blue (73)	Red (87)	Red (83)	Red (84)	NDVI (84)	Blue (88)	Red (61)	T3NDVI (97)
T4Blue (60)	T4Blue (80)	T5NDVI (85)	T3NIR (78)	T3Red (88)	T5NDVI (93)	T1Red (91)	T4Stu (87)	T5BG (88)	T6Red(80)	NIR (69)	NIR (77)	BG (55)	Blue (83)	Blue (83)	Gree (85)	BG (59)	T1Stu (95)
T4Gree (60)	T5Gree (86)	T6.BG (82)	T7Stu (76)	T5Blue (87)	T4NDVI (92)	T5Red (89)	T4Gre (83)	T5Blue (82)	T3BG (76)	Gree (56)	NDVI (71)	Gree (51)	NIR (77)	BG (82)	NDVI (84)	Gree (49)	T7NDVI (93)

^**α**^Image taking time from early April (T1) to October (T7) at about one month interval

^**β**^Abbreviations: Veg, vegetation; Non-Veg, non-vegetation; CropSys, Cropping systems; ATO, adult tree orchard; SUC, summer crops; WIC, winter crops; vegetation indices: NDVI, Stu (R/G) and B/G. and spectral bands: B, G, R and NIR.

It should be noted that, in fact, the VI values are ratios or combinations of SB, and that for many land use, a strong relationship exists between some bands and VI, as for the NIR and NDVI in the vegetation.

Regarding the independent variables or predictors that were used in the CRT DT analysis, it should be noted that a) CROPCLASS-2.0 automatically extracted for each parcel the average values of the SB of the corresponding images; b) CROPCLASS also automatically computed the VI used; and c) the DT software is a powerful tool that does not require extra costs and generates a very high amount of data in a cost-effective manner, selecting the most decisive independent variables regardless the number of independent variables. Therefore, it seems that the independent predictor variables that were used in our study are adequate and fit well with the objective. Furthermore, it is not logical to reduce the number of independent variables to perform similar studies.

### 5. Classification of unidentified parcels through prediction model testing

For the vegetation/non-vegetation discrimination and for the cropping systems discrimination the OA was 97% and 87% respectively. UA for most individual crops was 85% or higher, except for OAT (40%), likely because it’s morphological similarities with WHT. Averaged UA was 86%, 95% and 80% for ATO, SUC and WIC respectively (Table 8). The identification of parcels considering simultaneously all of the land uses produces an OA of 80%, and a UA that was 80% greater for crops such as the BNS, CKP and COT and lower for others, such as the CRN (89%) and WHT (69%) (Table 9). Furthermore, the OAT and POT were not correctly identified (0%) partly due to similarities with the WHT phenological pattern and to the low number of samples that were analyzed.

**Table 8:** Confusion matrix for the classification of unidentified adult tree orchard (ATO), summer crop (SUC) and winter crop (WIC) parcels by implementing SQL predictive models.

ATO								SUC								WIC							
	Obs.	CIT^α^	MFO	OLV	POP	Pred.	UA (%)^β^		Obs	COT	CRN	POT	SUN	Pred.	UA (%)^β^		Obs.	BNS	CKP	OAT	WHT	Pred.	UA (%)^β^
CIT	10	7 (0.75)		2 (0.9)	1 (1.0)	7	70	COT	10	10 (1.0)				10	100	BNS	8	8 (1.0)				8	100
MFO	8		8 (0.9)			8	100	CRN	9		9 (1.0)			9	100	CKP	8		7 (0.9)		1 (1.0)	7	87.5
OLI	18		1	16 (0.75)	1	16	89	POT	6			6 (1.0)		6	100	OAT	5			2 (1.0)	3 (1.0)	2	40
POP	8	1 (1.0)			7 (1.0)	7	87.5	SUN	10			2	8 (1.0)	6	80	WHT	29		1 (0.9)	1 (1.0)	27 1.0)	27	93
Obs.		8	9	18	9				35	10	9	8	8						8	8	3	31	
PA(%)^β^		87.5	89	89	78					100	100	75	100						100	87.5	677	87 2	
OA(%)^β^							86.4								94.2								88

^**α**^Abbreviations: BNS, broad beans; CKP, chickpeas; CIT, citrus orchards; COT, cotton; CRN, corn; MFO, Mediterranean forest; OAT, oat; OLV, olive orchards; POP, poplars grove; POT, potatoes; SUC, summer crops; SUN, sunflower; WHT, winter wheat;

^β^UA, User accuracy; PA: Producer Accuracy; OA, overall accuracy.

**Table 9:** Confusion matrix for unidentified parcel classification by applying individual crop SQL predictive models.

Crops	Parcels	BNS^α^	CIV	CKP	COT	CRN	MFO	OAT	OLV	CIT	POP	POT	SUN	WAT	WHT	YTO	Predicted parcels	UA^3^ (%)
BNS	8	8 (0.3)^β^															8	100
CIV	7		7 0.3)														7	100
CKP	8			8 (1.0)													8	100
COT	10				10 (0.9)												10	100
CRN	9				1 (0.92)	8(1.0)											9	89
MFO	8						8(0.89)										8	100
OAT	4							0							2 (1.0)	2 (1.0)	4	0
OLV	18								16 (0.7)							2 (1.0)	18	89
CIT	12						1 (0.9)		5 (0.7)	6 (0.7)							12	50
POP	8										8 (1.0)						8	100
POT	4	2 (0.32)										0			2 (1.0)		4	0
SUN	10	2 (0.32)											8 0.91)				10	80
WAT	7													7 (1.0)			7	100
WHT	29	6 (0.32)		2						1 (0.7)					20 (1.0)		29	69
YTO	14	2 (0.32)											2 (0.91)			10 (1.0)	14	71
Predicted	156	20	7	10	11	8	9	0	21	7	8	0	10	7	24	14	156	
PA ^$^ (%)		40	100	80	91	100	89	0	76	86	100	0	80	100	83	71		
OA^$^ (%)																	80	

^α^Abbreviations: Veg, vegetative; NonVeg, Non-vegetative; CropSys, Cropping systems; ATO, adult tree orchard; SUC, summer crops; WIC, winter crops; YTO, young trees orchards; BNS, broad beans; CKP, chickpeas; CIT, citrus orchards; COT, cotton; CRN, corn; MFO, Mediterranean forest; OAT, oat; OLV, olive orchards; POP, poplars grove; POT, potatoes; SUC, summer crops; SUN, sunflower; WHT, winter wheat; WIC, winter crops; YTO, Young Trees Orchards.

^β^In parenthesis: DT terminal node probability;

^$^UA, User accuracy; PA: Producer Accuracy; OA, overall accuracy.

## Discussion

Agriculture widely varies from one region to another mainly in the cropping systems and crop-growing calendar. Generally, the crop classification in any area is complex due to agronomic factors and farming decisions. Different crops can exhibit very similar developmental patterns and growth calendars, which is the case of autumn-sown wheat and oat. In addition, the same crop may be sown on different dates due to farmers’ decision. For example, in Mediterranean agriculture, due to the mild winter, sunflowers can be sown in mid-late winter or mid-spring. So that, the same crop may exhibits considerable differences in phenological patterns. Nevertheless, administrations for planning and subsidy policy actions need timely information on crop production at the regional level [1, 2, 3] and more precisely at the parcel level [4]. In our study area, twelve crops/cropping systems and two non-vegetative land uses were effectively classified at the census parcel levels through remote sensing images and the CROPCLASS procedure. A DT statistical analysis based on the SB and VI that were taken from each parcel at each remote image identified in our case study the vegetation and main cropping systems with approximately 100% and 94% correctly classified parcels. The adult tree plantation and winter cropping system parcels were somehow more efficiently discriminated (100%) than were the summer crops (91%) or young orchards (61%). Performing CROPCLASS through the CROPCLASS-2.0 software worked rapidly in an economically feasible manner and can be implemented in any geographical area. To perform the CROPCLASS procedure through conventional image processing would be highly time-consuming, requiring additional computer language programming skills, and would therefore not be viable for the practical use of administrations.

For crop classification at the census parcel level, several issues that are related to the remote image series should be considered. First, images with a high spatial resolution (i.e., 2 to 5 m of pixel) should be normally used to avoid or minimize crop-mixing pixels [4]. Second, georeferencing errors between the series of high spatial resolution images of the same scene are common and need to be corrected using hard-control points or the co-registration of each other to provide a given parcel with the same geographical references at any image [25]. Third, each image is instantaneity a given invariant features of an image series that does not normally provide the same reflectance or digital reading as that in the others images [31]; therefore, for classification purposes, multitemporal series of images should be calibrated or normalized. Absolute calibration can be achieved through the QUAC method [32] and FLAASH method [33], which use the solar position and weather calibration parameters. For agricultural scenes, an image time series can also be normalized using pseudo-invariant vegetative features, which avoid using solar and climate physical parameters [26].

A series of multitemporal images is usually needed to incorporate phenological observations for crop classification [3, 6, 10, 11, 15, 16]. Generally, in any agricultural scene, the number of remote images that are required for crop classification will vary with the crop diversity and the extension of the growing season. These concepts are obviously related to the climate and normally coincide with the freeze-free period or more precisely with mean temperatures greater than 4 or 6°C. The number and the timing of images were also important features that should be considered when applying the CROPCLASS procedure to any agricultural area. In our study, we took seven images throughout the growing season, approximately 1 every 3 to 4 weeks, and the crop classification results were satisfactory. Therefore, in general, taking an image every month during the active growing season can be a conservative recommendation for any region. However, our data also demonstrate good classification results from just analyzing 2 or 3 images at equidistance time intervals around the mid growing season. It should be noted that in Southern Spain, the winter is mild, and the growing period or freeze-free period lasts approximately 9 to 10 months. Therefore, the time interval between images taking can likely be extended. Furthermore, a working hypothesis is that in cold regions where the growing season can last only 4 or 5 months, the crop classification could tentatively be achieved with only 2 or 3 images distributed throughout the growing season.

The crop/land use statistical data were analyzed through DT similarly as previously described. and this has significant advantages, such as flexibility, easy interpretation of the model tree structure, computationally fast and no require assumptions regarding the distribution of the data [3, 18, 19, 21, 22, 23, 24]. In addition, the DT provides an SQL predictive model for each crop/cropping system, which is an additional original feature of CROPCLASS. It should be considered that the implementation of the SQL models of a specific crop should be used in the same area and using the same number of images that were taken at approximately the same time as those that were used to determine/generate the model.

CROPCLASS procedure meets additional advantages as follows. First, the census parcel is the unit for most administrative actions and CROPCLASS provide record for each census parcel. Second, administrations require a defined crop classification method, almost fully relying on remote sensed images that were automatically or semi-automatically executed, and requiring a reduced ground-visit work effort. Third, the predictive models for each crop/cropping system are likely to be used for the same area in subsequent years if the images were taken on about the same dates. This use is based on the true assumption that in each geographical area, the diversity of the crops and the crop calendar remain about the same throughout the years. Similarly, Zhong et al. (2014; [16]) mapped crops in multiple years using training data that were limited to a single year based on phenological metric and vegetation indices. So that, the phenology or crop growth stages will approximately coincide, as the images were taken at about the same time in different years; therefore, the predictive models that were determined for one year with similar timings could tentatively be used in subsequent years. Similarly, the parcel SB and VI data matrix from a new year can be added to the previous years, thereby constructing a multi-years matrix data. The field work and image taking of this study lasted only for one year; therefore, the verification of these assumptions is out of its scope. However, the assumptions that CROPCLASS data and models for each specific area can be used in subsequent years can be accepted due to the specificity of the crop diversity and crop calendar for any region, which is repeated throughout the years.

## Conclusions

A novel method named CROPCLASS for census parcel classification from multitemporal remote images was developed for use in any agricultural and forestry area of the world. This new procedure defines the census parcels in every scene, extracts the SB and VI values from each parcel of each image, and monitors the Decision Tree analysis of the matrix data. The independent predictor variables that were used in our study are adequate and fit well with the objective. Necessarily, this method also includes the ground-truth visit and identification of a reduced number of parcels in at least the first year of study. The CROPCLASS procedure also monitors the SQL prediction models that are generated in the DT analysis, implementing these models to identify the crops of unknown parcels. We validated CROPCLASS using a series of GeoEye-1 satellite images of one scene. Implementing the CROPCLASS procedure through conventional image processing is time consuming and requires computer language skills. The software CROPCLASS-2.0 executes the CROPCLASS methodology semi-automatically in an economically feasible manner and can be implemented for any agricultural region.

## Supporting Information

S1 Appendix Partial view of the Structured Query Language (SQL) models to identify the winter crops. (DOCX)
