# Supplementary material for: Census Parcels Cropping System Classification from Multitemporal Remote Imagery: A Proposed Universal Methodology
Source: PLoS One. 2015 Feb 17;10(2):e0117551. doi: 10.1371/journal.pone.0117551 (PMC4331289; doi:10.1371/journal.pone.0117551)
Supplement: S1 Appendix — (DOCX) [file pone.0117551.s001.docx]

*Supporting file for PLoS ONE, final version 08 January 2015*

*CROPCLAS a*rticle: Census parcels cropping system classification from multitemporal remote imagery: a proposed universal methodology

**Supporting Information**

**Appendix S1.** Partial view of the Structured Query Language (SQL) models to identify the winter crops.

/* Node 7 */.

UPDATE <TABLE>

SET nod_001 = 7, pre_001 = 'oat', prb_001 = 1.000000

WHERE (((NOT(T1.NIR IS NULL) AND (T1.NIR <= 4324)) OR (T1.NIR IS NULL) AND ((NOT(T5.BG IS NULL) AND (T5.BG <= 0.6745000000000001)) OR (T5.BG IS NULL) AND ((NOT(T1.NDVI IS NULL) AND (T1.NDVI <= 0.7124999999999999)) OR (T1.NDVI IS NULL) AND ((NOT(T1.Red IS NULL) AND (T1.Red > 750)) OR (T1.Red IS NULL) AND ((NOT(T1.Stu IS NULL) AND (T1.Stu > 0.8100000000000001)) OR (T1.Stu IS NULL) AND ((NOT(T4.BG IS NULL) AND (T4.BG <= 0.6775)) OR (T4.BG IS NULL) AND ((NOT(T1.Gree IS NULL) AND (T1.Gree > 927)) OR (T1.Gree IS NULL) AND ((NOT(T5.Blue IS NULL) AND (T5.Blue <= 791)) OR (T5.Blue IS NULL) AND ((NOT(T1.Blue IS NULL) AND (T1.Blue > 613.5)) OR (T1.Blue IS NULL) AND ((NOT(T6.BG IS NULL) AND (T6.BG <= 0.631)) OR (T6.BG IS NULL) AND ((NOT(T4.Blue IS NULL) AND (T4.Blue <= 575)) OR (T4.Blue IS NULL) AND ((NOT(T4.Gree IS NULL) AND (T4.Gree <= 853)) OR (T4.Gree IS NULL) AND ((NOT(T7.BG IS NULL) AND (T7.BG <= 0.7)) OR (T7.BG IS NULL) AND ((NOT(T6.Stu IS NULL) AND (T6.Stu > 1.7105)) OR (T6.Stu IS NULL) AND ((NOT(T5.Gree IS NULL) AND (T5.Gree <= 1144.5)) OR (T5.Gree IS NULL) AND ((NOT(T2.NDVI IS NULL) AND (T2.NDVI <= 0.628)) OR (T2.NDVI IS NULL) AND ((NOT(T3.NIR IS NULL) AND (T3.NIR <= 3163)) OR (T3.NIR IS NULL) AND ((NOT(T3.Blue IS NULL) AND (T3.Blue <= 861.5)) OR (T3.Blue IS NULL) AND

…………………………………………………………………………..

……………………………………………… R (T1.NDVI IS NULL) AND ((NOT(T1.Red IS NULL) AND (T1.Red <= 1153)) OR (T1.Red IS NULL) AND ((NOT(T2.BG IS NULL) AND (T2.BG <= 0.4255)) OR (T2.BG IS NULL) AND ((NOT(T2.Red IS NULL) AND (T2.Red <= 1180.5)) OR (T2.Red IS NULL) AND ((NOT(T3.Red IS NULL) AND (T3.Red <= 1642.5)) OR (T3.Red IS NULL) AND ((NOT(T1.Stu IS NULL) AND (T1.Stu <= 0.8425)) OR (T1.Stu IS NULL) AND ((NOT(T2.Blue IS NULL) AND (T2.Blue <= 238)) OR (T2.Blue IS NULL) AND ((NOT(T5.Red IS NULL) AND (T5.Red > 1779.5)) OR (T5.Red IS NULL) AND ((NOT(T6.BG IS NULL) AND (T6.BG <= 0.6174999999999999)) OR (T6.BG IS NULL) AND ((NOT(T2.NIR IS NULL) AND (T2.NIR <= 4127.5)) OR (T2.NIR IS NULL) AND ((NOT(T2.Stu IS NULL) AND (T2.Stu > 1.353)) OR (T2.Stu IS NULL) AND ((NOT(T3.NDVI IS NULL) AND (T3.NDVI <= 0.3695)) OR (T3.NDVI IS NULL) AND ((NOT(T3.NIR IS NULL) AND (T3.NIR <= 3682.5)) OR (T3.NIR IS NULL) AND ((NOT(T4.NIR IS NULL) AND (T4.NIR <= 2241.5)) OR (T4.NIR IS NULL) AND ((NOT(T5.NIR IS NULL) AND (T5.NIR <= 2832)) OR (T5.NIR IS NULL) AND ((NOT(T6.NIR IS NULL) AND (T6.NIR <= 2417.5)) OR (T6.NIR IS NULL) AND ((NOT(T6.Red IS NULL) AND (T6.Red <= 1661)) OR (T6.Red IS NULL) AND ((NOT(T1.NIR IS NULL) AND (T1.NIR > 3887.5)) OR (T1.NIR IS NULL) AND ((NOT(T2.NDVI IS NULL) AND (T2.NDVI > 0.521)) OR (T2.NDVI IS NULL) AND ((NOT(T4.BG IS NULL) AND (T4.BG > 0.637)) OR (T4.BG IS NULL) AND (NOT(T5.BG IS NULL) AND (T5.BG > 0.6525000000000001)))))))))))))))))))))))))))))))))))))))))))))))))));

**Supporting Information Legends**

**Appendix S1.** Partial view of the Structured Query Language (SQL) models to identify the winter crops

.
